# 

**Published:** 1913-01-04

**Authors:** 


### Books Received.

University of London Press: "Diseases of Women," by T. G. Stevens, M.D.; "Diseases of the Eyes," by G. Devereux Marshall; "Medical Diseases of Children," by T. Rowland C. Whipham; "Treatment After Operation," by Wm. Turner and E. Rock Carling.

T. C. and E. C. Jack: "Hypnotism," by A. M. Hutchison; "Marriage and Motherhood," by H. S. Davidson, M.B.

W. J. Gray: "The Writers' and Artists' Year Book, 1913."

Mr. Arthur Skeffington, of 49 Ulundi Road, Blackheath, the inventor of patent Invalid Lifters, has been awarded a gold medal, the highest award, at the recent National Winter Sports Exhibition, Vienna, and also grand prize and gold medal at the International Exhibition lately held in Milan. Mr. Skeffington's Invalid Lifters have often been described in The Hospital, and his latest success will come as no surprise to institutional managers.

### Buildings Contemplated.

Lancaster.—The Lancashire Asylums Board has received sanction to a £30,000 loan for the erection of villas for private patients at Lancaster Asylum.

Newton Abbot.—Juvenile reception wards and infectious isolation accommodation are projected by Newton Abbot Guardians.

Winsley (Bath).—Mr. W. S. Skinner, of Bristol, is architect for extension of the kitchen department at Winsley Sanatorium; a tender of £3,548 has been received, also one of £1,164 for men's chalets, and £180 for women's ward alterations.

Ruthin.—The Ruthin Guardians have decided to erect a new infirmary and cottage homes on a site adjoining the workhouse.

Wolverhampton.—It is proposed to provide, at an estimated cost of £54,100, ward blocks or pavilions, and other buildings are also projected. An appeal is being made by the governors of the Wolverhampton and Staffordshire General Hospital for the necessary capital to warrant proceeding.

Sydney.—An enlargement of Sydney General Hospital is projected at an estimated cost of £150,000.

Stockport.—£11,000 has been raised for infirmary extension as a memorial to the late King. The work has been inaugurated by the laying of a foundation-stone to the nurses' new home.

Barnet.—Ten designs, submitted in competition, have been considered by the Guardians for a children's home in connection with the workhouse. Premiums of twenty-five, fifteen, and ten guineas were awarded respectively to the designs under mottoes "Economy," "Compactum," and "Fiat Lux."

Blaina.—An extension to the cottage hospital is projected at an estimated cost of £2,500.

Bootle.—Plans by the Borough Engineer, Mr. B. J. Wolfenden, for works to the smallpox hospital at Maghull and infectious diseases hospital, at respective costs of £2,004 and £1,749, have been the subject of a Local Government Board inquiry.

### Appointments.

Mr. T. W. R. Strode has been appointed as junior assistant medical officer at the Manor Mental Hospital, Epsom.

Dr. B. D. Crichton, of Dublin, has been appointed assistant house surgeon at the West Bromwich District Hospital.

Mr. Howard H. C. Dent, M.B., F.R.C.S., L.R.C.P., has been elected an honorary surgeon to the Wolverhampton and Staffordshire General Hospital. Mr. Dent has held the post of honorary assistant surgeon since January 1902.

Mr. H. Hyslop Thomson, M.D., D.P.H., tuberculosis officer for Newport and East Monmouthshire, and formerly medical superintendent of the Liverpool Sanatorium, has been appointed tuberculosis officer for the county of Hertford.

